# Pledget-Armed Sutures Affect the Haemodynamic Performance of Biologic Aortic Valve Substitutes: A Preliminary Experimental and Computational Study

**DOI:** 10.1007/s13239-016-0284-8

**Published:** 2016-11-21

**Authors:** Claudio Capelli, Chiara Corsini, Dario Biscarini, Francesco Ruffini, Francesco Migliavacca, Alfred Kocher, Guenther Laufer, Andrew M. Taylor, Silvia Schievano, Martin Andreas, Gaetano Burriesci, Claus Rath

**Affiliations:** 1 UCL Institute of Cardiovascular Science, and Great Ormond Street Hospital for Children, NHS Foundation Trust, 30 Guilford Street, London, WC1N 1EH UK; 2 Laboratory of Biological Structure Mechanics, Department of Chemistry, Materials and Chemical Engineering ‘Giulio Natta’, Politecnico di Milano, Milan, Italy; 3 Department of Cardiac Surgery, Medical University of Vienna, Vienna, Austria; 4 Department of Mechanical Engineering, UCL, London, UK; 5 Fondazione Ri.MED, Bioengineering Group, Palermo, Italy; 6 Division of Anatomy, Center for Anatomy and Cell Biology, Medical University of Vienna, Vienna, Austria

**Keywords:** Valve prostheses, Pledgets, Hydrodynamics, Standard test, Computational fluid dynamics

## Abstract

Surgical aortic valve replacement is the most common procedure of choice for the treatment of severe aortic stenosis. Bioprosthetic valves are traditionally sewed-in the aortic root by means of pledget-armed sutures during open-heart surgery. Recently, novel bioprostheses which include a stent-based anchoring system have been introduced to allow rapid implantation, therefore reducing the duration and invasiveness of the intervention. Different effects on the hemodynamics were clinically reported associated with the two technologies. The aim of this study was therefore to investigate whether the differences in hemodynamic performances are an effect of different anchoring systems. Two commercially available bio-prosthetic aortic valves, one sewed-in with pledget-armed sutures and one rapid-deployment, were thus tested in this study by means of a combined approach of experimental and computational tools. *In vitro* experiments were performed to evaluate the overall hydrodynamic performance under identical standard conditions; computational fluid dynamics analyses were set-up to explore local flow variations due to different design of the anchoring system. The results showed how the performance of cardiac valve substitutes is negatively affected by the presence of pledget-armed sutures. These are causing flow disturbances, which in turn increase the mean pressure gradient and decrease the effective orifice area. The combined approach of experiments and numerical simulations can be effectively used to quantify the detailed relationship between local fluid-dynamics and overall performances associated with different valve technologies.

## Introduction

Aortic stenosis (AS) is the most common valvular heart disease in industrialized countries, and its impact on public health is expected to increase due to the aging population.^8^ Surgical aortic valve replacement with either a mechanical or a biological valve is considered the golden standard of care in patients with severe AS.^34^,^36^ In elderly patients, sewed-in, stented bioprosthetic valves are the most commonly implanted devices.^25^ They typically consist of a plastic and/or metal frame, coated with fabric or tissue, holding three leaflets made of xenograft soft tissue. At the base of the valve, a sewing ring covered in fine fabric is attached. For the implantation, pledget-armed sutures are commonly used to secure the valve in either an intra- or supra-annular position.^1^,^3^,^32^ To decrease the surgical trauma of the implantation procedure, and the associated peri-operative risks, less invasive procedures have been explored.^12^ Hence, rapid-deployment valve systems have been recently introduced to the clinical arena.^15^,^16^ Currently, two systems are commercially available in this group of devices, also generally known as “sutureless valves”: Sorin Perceval (Sorin Group, Italy), and Edwards Intuity (Edwards Lifesciences, CA). Both are inserted either *via* full- or mini-sternotomy or *via* right anterior thoracotomy. These valves can be rapidly implanted by the dilatation of a stent frame to ensure the anchoring of the prosthesis. Radial pressure of the stent frame should ensure a safe contact between the device and the debrided aortic annulus with no requirement for pledget-armed sutures. Clinical studies evaluating these valves suggested safe implantation with associated reduced duration of cross-clamp and cardiopulmonary bypass time compared to conventional valves.^26^ In addition, the devices seem to show excellent early hemodynamic outcome.^1^,^26^ Such improved hemodynamic performance might be related to the absence of pledget-reinforced sutures.^1^ A recently published *in vitro* study supports this hypothesis.^30^ However, in current clinical studies, the influence of the suture technique is still under debate.^29^,^33^

The assessment of the hemodynamic performance associated with heart valve substitutes has been extensively standardized (ISO 5840:2009). In particular, standardized experiments have been defined to test novel valves,^10^,^31^ to compare available devices^21^ or, as in the context of this study, to measure the influence of suture techniques on the bioprosthesis performance.^35^ Furthermore, computational modelling has become an increasingly recognized tool for analysing the flow conditions associated with the implantation of valve prostheses. Computational fluid dynamics (CFD) tools can in fact provide detailed information about flow field and mass transport in complex geometries and contribute to highlighting mechanisms of pathogenesis.^35^ In this context, Bluestein and colleagues investigated the complex hemodynamics of a bileaflet mechanical heart valve through unsteady CFD simulations.^5^ Streamlines visualization revealed vortex shedding in the wake of the valve leaflets during the deceleration phase, and particle paths showed the presence of platelets within the shed vortices. This hemodynamic phenomenon might explain platelet aggregation and, consequently, the development of free emboli carried downstream by the shed vortices. Sirois and Sun examined the risk for shear-induced hemolysis and platelet activation using a numerical bioprosthetic valve model.^28^ From the resulting hemodynamic quantities such as flow velocity, wall shear stress at the valve leaflets and turbulent losses, they found that the folded geometry of the valve leaflets in the fully open position might have an impact on the hemodynamic performance of the bioprosthetic valve. Another computational study compared the hemodynamics and thrombogenicity of two bileaflet mechanical heart valves using CFD blood models interacting with the moving valve leaflets.^14^ Pressure gradients and velocity patterns were comparable between the two valve models, whereas differences were noted in the platelet stress accumulation during the regurgitation flow phase. These findings suggested that the different designs of the two valves might lead to different extents of platelet activation. More recently, a testing approach, which combines *in vitro* and *in silico* tools^10^,^38^ has been suggested to evaluate the effects of small valve design variations by not only recording the overall performance, but also highlighting the flow disturbances which might explain any difference.

This approach seems to be particularly indicated to characterize the influence of mounting techniques, such as the presence of pledget-armed sutures. Hence, the aim of this study is to compare the performance of one bioprosthesis implanted with standard surgical fixation technique against its rapid deployed counterpart. The quantified effect of different mounting elements will help to understand the role played by the surgical implantation technique and to improve design of future valve substitutes according to the detected hemodynamic influence.

## Materials and Methods

**Figure 1:**
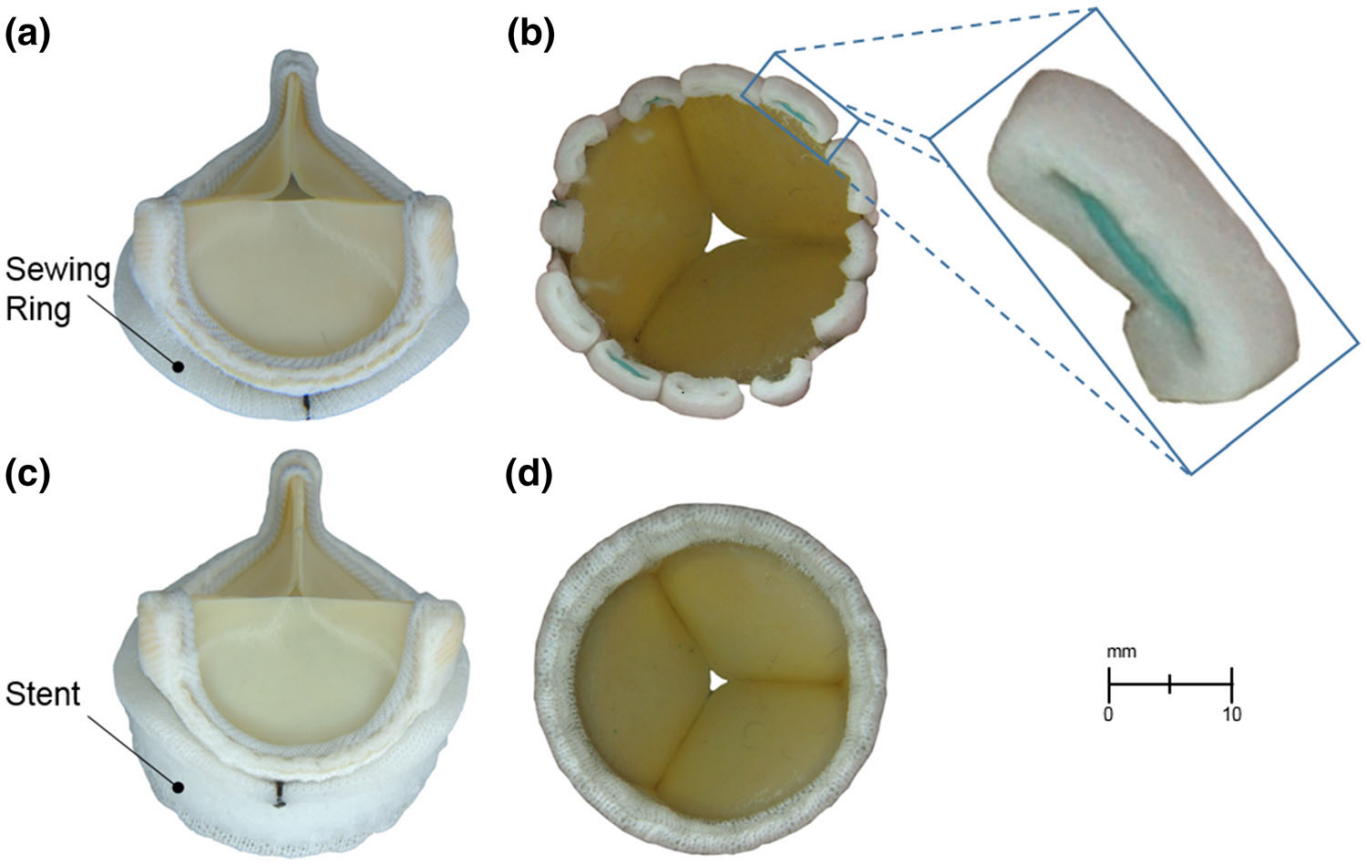
The two valves tested in this study: Carpentier-Edwards PERIMOUNT Magna Ease Aortic Heart Valve (Magna) and Edwards Intuity Valve System (Intuity). (a) Front view of Magna; (b) bottom view of stitched Magna including the pledget-armed sutures (magnified); (c) front view of Intuity; and (d) bottom view of Intuity. At the bottom part of the Intuity valve it is possible to distinguish the polyester covered stent frame to be expanded for ensuring the anchoring of the valve.

The hemodynamic performance of two bioprosthetic valves were compared in this study, namely the sewed-in bioprosthetic aortic heart valve Carpentier-Edwards PERIMOUNT Magna Ease Aortic Heart Valve (Magna) and the rapid-deployment bioprosthetic aortic heart valve Edwards Intuity Valve System (Intuity). These two devices are based on the same valve platform. A stainless steel frame holds together three leaflets made of fixed bovine pericardium. Both valves are designed to be placed in the same, supra-annular position, and differ only in their mounting system. While the Magna valve is sewed into the aortic root with pledget-armed sutures, the Intuity valve system is anchored with a polyester sealing cloth-covered, balloon-expandable, stent frame at the inflow aspect (Fig. 1).

The hemodynamic properties of Magna and Intuity were investigated by means of both experiments and CFD simulations, which will be described in the following sub-sections.

### Experimental Testing

*In vitro*, two valve samples, one Magna and one Intuity were tested. The nominal size of the devices was 23 mm (internal diameter 22 mm, profile height 15 mm, external sewing diameter 28 mm). The Magna valve was tested in two configurations with (Magna_Pledgets) and without pledget-armed sutures (Magna). The valves were mounted on silicon rubber holders, which were specifically designed and manufactured for this study by moulding technique. The holders for testing Intuity and Magna were identical. The holder to test Magna_Pledgets was embedded with polyester to avoid the tearing of the sutures. For fixation, polyester, non-absorbable, braided, coated sutures with 6 × 3 × 1.5 mm firm PTFE pledgets were used (CV Pass, PremiCron, Braun, Germany). These sutures have oval shaped pledgets with reduced material and they were chosen to minimize potential outflow-tract obstruction.

Fluid dynamic performance was assessed by means of a hydro-mechanical pulse duplicator (ViVitroLabs Inc., Canada) which simulates the function of the ventricle to generate pulsatile flow through the heart valve substitute. This system,^27^ designed to comply with ISO 5840 and FDA requirements, was able to replicate a physiological range of operating conditions. The bioprostheses were tested in the aortic position with a mechanical control valve (Sorin Allcarbon valve) mounted in the mitral position. Saline solution (0.9% w/v of NaCl) was used in the tests. Pressure measurements were acquired using Millar Mikro-Tip catheter transducers (Millar Inc., Huston, TX). Flow was measured using an electromagnetic flow probe (Carolina Medical Electronics, East Bend, USA) placed in the aortic position (upstream the valve). Hydrodynamic parameters were acquired at increasing cardiac outputs between 2 and 7 L/min, with a mean arterial pressure of 100 mmHg, a fixed heart rate of 70 beats/min (bpm) and systole occupying 35% of the cycle. The measurements were collected during ten cardiac cycles and analysed in the VSI software package (VSI, ViVitro, Victoria, BC, Canada). The leaflets kinematics were visualised using a high-speed camera. For each sample, a total of ten measurements were recorded and consequently analysed for statistical significance using one-way ANOVA test.^28^ Two-sample *t*-tests assuming unequal variances were performed to verify the significant difference (*p* < 0.05) of mean pressure gradient and effective orifice area (EOA) for varying cardiac outputs. EOA was calculated according to the following definition [1]:

1 $$ {\text{EOA}} = \frac{{q_{{{\text{V}}_{\text{RMS}} }} }}{{51.6\sqrt {\frac{\Delta p}{\rho }} }} $$

where EOA is the effective orifice area in square centimetres, $q_{{{\text{V}}_{\text{RMS}} }}$ is the root mean square forward flow in ml/s, $\Delta p$ is the mean pressure difference in mmHg, and *ρ* is the density of the test fluid in g/cm^3^.

### Computational Analyses

**Figure 2:**
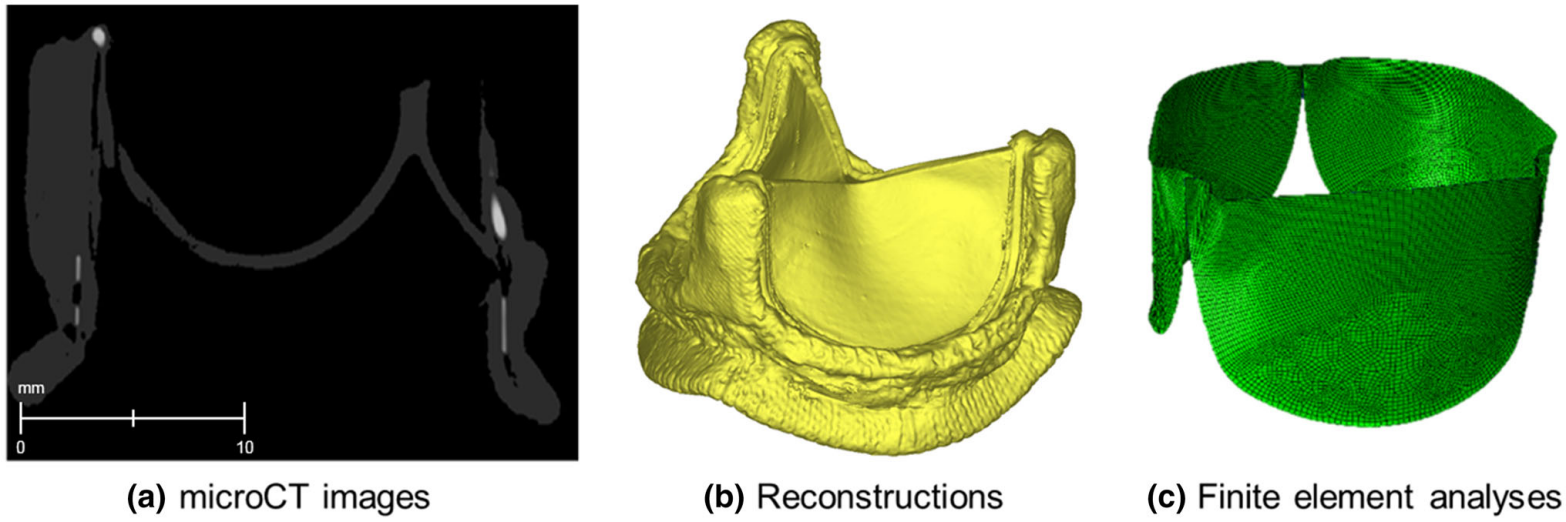
Phases of the modelling of the valve leaflets: (a) microCT images were acquired with high resolution (40.4 *μ*m); (b) a 3D model of the bioprosthesis was reconstructed following the segmentation of the images; (c) open configuration of the leaflets as obtained by finite element analyses.

*In silico* CFD models were set up to replicate the systolic forward flow phase of the tests at 5 L/min, with the valve leaflets in open configuration. The 3D models reproduced the main geometry of the experimental apparatus. The fluid domains included the pipe connecting the ventricular chamber to the valve holder (i.e., inlet pipe), the valve holder, the valve leaflets and the aortic root (i.e., the outlet pipe). This volume was selected to calculate flow and pressure differences across the valves. The inlet and outlet pipes were 40.0 mm in length and 28.0 mm in diameter. The design of the aortic root replicated the size and shape of the glass element used in the experiments (44.7 mm in height, 33.2 mm in diameter at the bottom and 28.8 mm at the top). The fluid domain also included the fluid pool surrounding the valve leaflets in open configuration. The geometry of the valve leaflets was derived according to the following steps (visualized in Fig. 2). First, a microCT acquisition of Magna and Intuity valves was performed with a spatial resolution of 40.4 *μ*m. The microCT scanner was a Metris X-Tek HMX ST 225 CT (Nikon) available at the Natural History Museum (London, UK). The analysis of the images confirmed that the design of frame and leaflets were identical for the different devices. Second, a CAD model of the device with closed leaflets was reconstructed by segmenting the acquired images with commercial software (Mimics Research 17.0, Materialise). Third, the opening of the leaflets was simulated with a preliminary finite element analysis. Each leaflet was modelled with 7424 quadrangular shell elements (a thickness of 0.5 mm, a mass density of 1120 kg/m^3^, a linear elastic modulus of 6 MPa, and a Poisson ratio of 0.45). A pressure of 120 mmHg was applied on the leaflets to cause their opening. Simulations were performed using commercial software (Abaqus 6.14, Dassault Systemes). Finally, the obtained open configuration of the leaflets was compared to images acquired during the experiments of both the valves at 5 Lpm and 70 bpm. Snap shots of the systolic phase of all the valves were in fact recorded by a high-speed camera (Nikon 1 V1, Nikon, Japan) placed over the aortic root coaxial with the valve and superimposed to the model of the leaflets. A correction (i.e., adjustment of the opening pressure) was applied to the model of the leaflets to match the captured orifice area.

**Figure 3:**
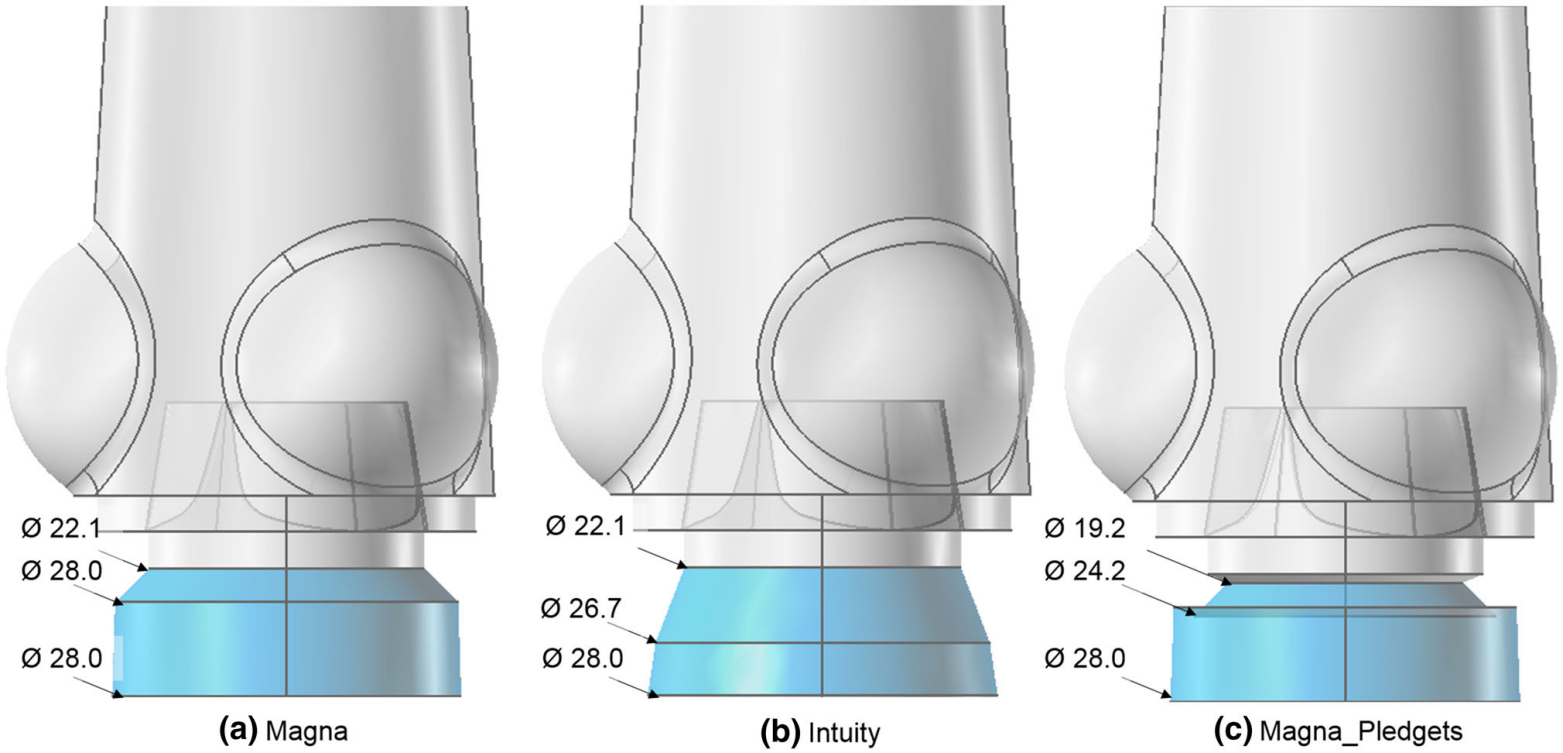
Anchoring system (light blue) and aortic root with valve leaflet (translucent grey) included in the model of computational fluid dynamics with highlighted the different sizes [mm] of the three settings: (a) Magna; (b) Intuity and (c) Magna_Pledgets.

The three different anchoring systems were modelled as depicted in Fig. 3 (light blue): (a) for the Magna by including a cylinder with a 28.0 mm diameter to represent the internal dimensions of the valve holder; (b) for the Intuity, by adding a truncated cone with a 10.4 mm slant height and to mimic the presence of the anchoring stent and its connection to the holder; and (c) for the Magna_Pledgets by subtracting a toroidal volume from the 3D domain at the level of the upper conical part of the valve holder to model the pledgets used for suturing the valve into it.

**Figure 4:**
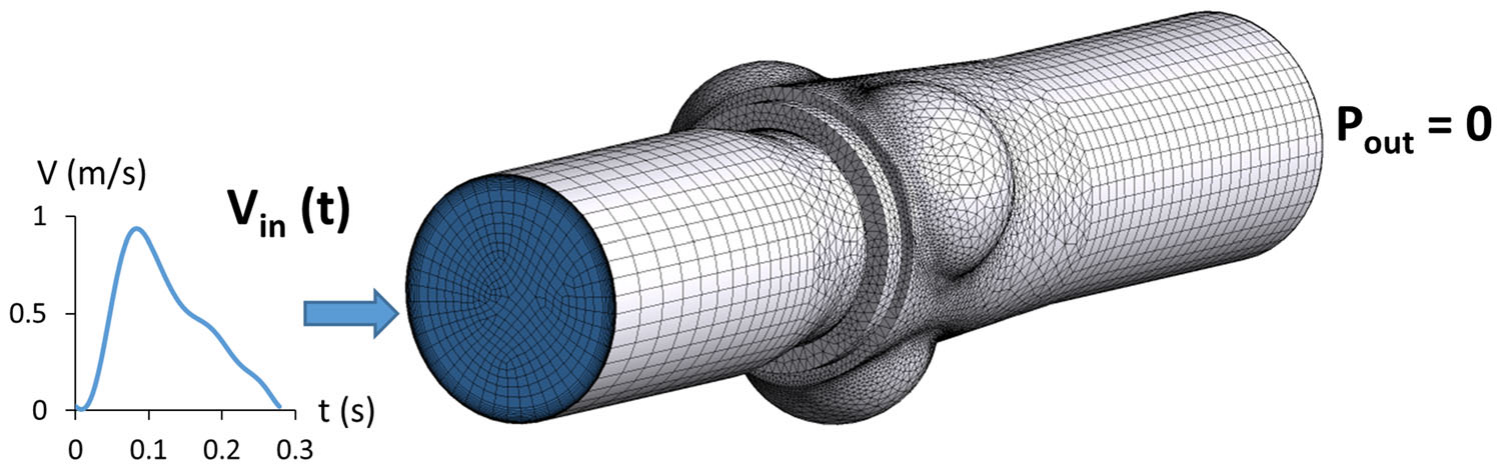
CFD model of Intuity with mesh and scheme of boundary conditions: at the inlet section, a time-varying velocity flat profile was applied as derived from experimental measurements; at the outlet, pressure was kept constantly equal to 0 mmHg.

The obtained geometries were meshed with ANSYS Meshing (Ansys Inc., Canonsburg, PA) software, using hexahedral elements for the pipe components and tetrahedral elements for the central part including the valve. For the latter, a curvature size function was adopted in order to refine the grid size according to the elements’ curvature on edges and faces. The near-wall regions were discretized using prism inflation layers defined by: maximum number of layers = 14, first layer thickness = 3 × 10^−5^ m and growth rate = 1.2. Following mesh sensitivity tests, based on the evaluation of mean pressure changes at the inlet, approximately 1.2 million elements were used as the grid density for all geometries, with maximum and minimum face sizes of 4 × 10^−3^ and 7 × 10^−6^ m, respectively. Figure 4 shows the mesh and the boundary conditions of the Intuity model as an example.

The fluid was modelled as Newtonian, homogeneous and incompressible with density = 1000 kg/m^3^ and dynamic viscosity = 0.001 kg/m/s. A time-varying velocity profile was applied at the inlet section, whereas constant zero pressure was imposed at the outlet. Moreover, the pipe walls were assumed as being rigid and a no-slip boundary condition was prescribed. The inlet velocity tracing for each model was reconstructed from the experimental flow signal measured in the systolic window. In Fig. 4 the waveform imposed to the Intuity model inlet is given as an example. The velocity profile was modelled as flat, since the section where flow was measured in the experimental set up was only 1.1 diameters downstream the entrance of the inlet pipe. It is worth noting that, by neglecting wall compliance, the pressure drop across each model was independent of the imposed outlet pressure. Therefore, it was calculated as equal to the time-varying inlet pressure. Unsteady particle tracking was used to quantify the particles residing in the 3D domain and the particle residence time (PRT), i.e., the time that has elapsed since the particles at any location throughout the domain entered the lumen.^19^ The inlet of the model was uniformly seeded with 4000 particles which were released every 1/70th of the cardiac cycle. Particles were assumed to be massless, thus passively following the flow field, and diffusion was neglected given the short residence time of the particles in this computational domain. Furthermore, adhesion between particles or between particles and side-walls was not accounted for. A 5th order Runge–Kutta integration algorithm was used as the tracking scheme with a maximum error tolerance of 10^−5^.

From a fluid-dynamic perspective, the simulated experimental conditions were characterized by high peak velocity (i.e., 1.3–1.4 m/s) through the valve, a sudden change in diameter due to the presence of the valve, as well as pulsatile conditions (heart rate = 70 bpm). Hence, the expected high Reynolds and Womersley numbers warranted the choice of using a turbulence model in order to handle the complex flow field characterizing the investigated 3D domain. The shear stress transport *k*–*ω* turbulence model was adopted.^22^ A turbulent intensity of 1% and length scale of 6.3 × 10^−4^ m were applied at the inlet boundary. In a boundary layer flow, it is recommended^2^ that a non-dimensional magnitude, *Y* ^+^, be sufficiently small (i.e., <4). *Y* ^+^ is defined as:

2 $$ Y^{ + } = \frac{{\rho  u^{*} y}}{\mu } $$

where *ρ* is the fluid density, u* is the friction velocity at the nearest wall (defined as the square root of the ratio between the wall shear stress and the fluid density), *y* is the distance from the nearest wall and *μ* is the fluid dynamic viscosity. Computer simulations were run using ANSYS Fluent (Ansys Inc., Canonsburg, PA). The SIMPLE algorithm was set as the pressure–velocity coupling scheme, with second-order spatial discretization for solving the Navier–Stokes and turbulence equations. A second order implicit method was applied for the transient formulation, with a time step of 4 × 10^−4^ s, which was determined by the widely known Courant–Friedrichs–Lewy condition. For each model, three systolic cycles were run on a parallel cluster compute node, with two Quad-Core Intel Xeon E5620 processors, requiring about 7 h for one cycle. Results from the last cardiac cycle were used to calculate pressure drops and PRT, as well as to display colour maps of pressure, velocity and wall shear stress (WSS). Other investigated quantities were the turbulent kinetic energy (TKE) and the localized normalized helicity (LNH). The TKE is the kinetic energy per unit mass^18^ associated with the fluctuating component $\tilde{\varvec{v}}(\varvec{x},t)$ of the velocity field, and is defined as follows:

3 $$ {\text{TKE}}\left( {\varvec{x},t} \right) = \frac{1}{2}\rho \left[ {\tilde{v}_{1}^{2} \left( {\varvec{x},t} \right) + \tilde{v}_{2}^{2} \left( {\varvec{x},t} \right) + \tilde{v}_{3}^{2} (\varvec{x},t)} \right] $$

where *ρ* is the flow density and $\tilde{v}_{i} (\varvec{x},t)$ denotes the *i*th component of $\tilde{\varvec{v}}(\varvec{x},t)$. TKE was computed in order to study turbulence in the 3D domain. LNH is a measure of the alignment/misalignment of the local velocity and vorticity vectors, its value ranges from −1 to 1 and its sign indicates the direction of rotation of helical structures.^24^ It is defined as the cosine of the angle between the velocity ($\varvec{v}(\varvec{x},t)$) and the vorticity ($\varvec{w}(\varvec{x},t)$) vectors of the flow in each point $\varvec{x}$ of the 3D domain and per each time point *t* according to:

4 $$ {\text{LNH}}\left( {x,t} \right) = \frac{{\varvec{v}(\varvec{x},t) \cdot \varvec{w}(\varvec{x},t)}}{{\left| {\varvec{v}(\varvec{x},t)} \right|\left| {\varvec{w}(\varvec{x},t)} \right|}} $$

LNH values close to −1/+1 indicate a marked alignment of the velocity and vorticity vectors, which is the necessary condition for highly helical flow. Points in the domain with the same LNH absolute values and opposite signs reveal the presence of counter-rotating helical structures of the same intensity. LNH iso-surfaces were computed for a better visualization of the complex flow patterns developing downstream the valve.

## Results

### Experimental Testing

The experiments provided a comparative assessment of the hydrodynamics of the three valve configurations tested.

**Figure 5:**
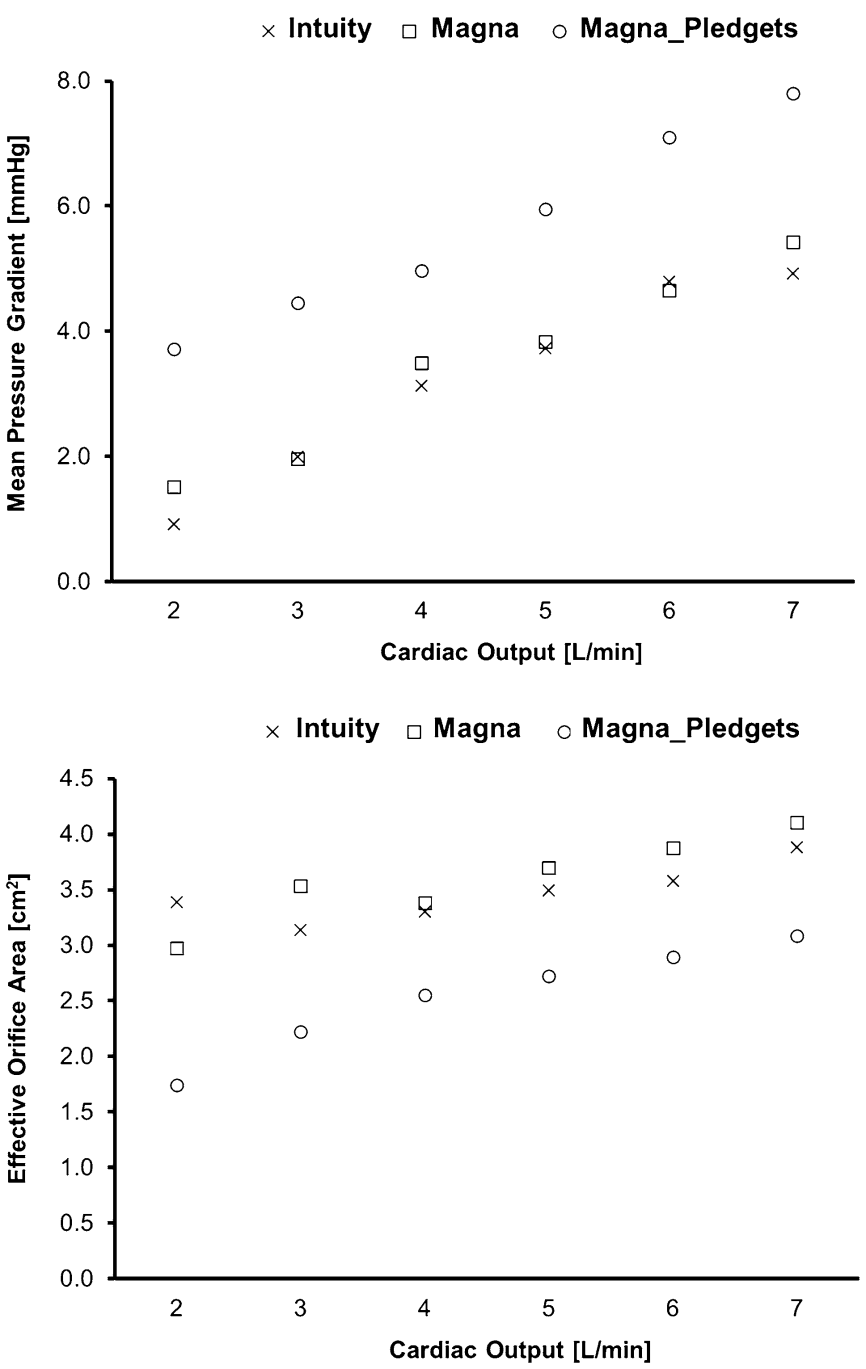
Graphs of mean pressure gradient (top) and effective orifice area (bottom) for increasing cardiac outputs of the three valves tested.

The results reported in the graphs of Fig. 5a show how the trends of mean pressure gradient (MPG) and effective orifice area (EOA) did not differ significantly between the Intuity and the Magna valves (*p* = 0.80, *p* = 0.51, respectively). For varying cardiac outputs, the Magna_Pledgets had significantly higher MPGs than the Intuity (*p* < 0.05) and the Magna valve (*p* < 0.05). In particular, the Magna_Pledgets valve showed an average increase of 2.18 mmHg in terms of MPG compared to the Magna tested without pledgets and an average increase of 2.41 mmHg compared to the Intuity. The measured EOA (Fig. 5b) was found to be equivalent (*p* = 0.51) for Magna and Intuity with an average difference of 0.13 cm^2^. The Magna_Pledgets had a significantly reduced EOA in all the tested conditions compared to both Intuity and Magna (*p* < 0.05). Peak pressure gradients measured at 5 L/min were 24.70, 24.13, and 32.62 mmHg for Intuity, Magna and Magna_Pledgets, respectively. Regurgitant fractions at 5 L/min, expressed as percentage of the stroke volume, were 3.44 ± 1.94 for the Intuity, 4.93 ± 1.53 for the Magna and 7.08 ± 3.86 for the Magna_Pledgets.

### Computational Analyses

The mean pressure drops obtained from CFD simulations were 1.33 mmHg (peak 27.9 mmHg) for the Intuity model, 1.58 mmHg (peak 25.5 mmHg) for the Magna and 1.92 mmHg (peak 30.9 mmHg) for the Magna_Pledgets.

For each valve, the local fluid dynamics was analysed at three time points during the simulated cycle, namely peak systole (or mid-acceleration i.e., *t*_1_ = 40 ms after the beginning of systole), peak flow (*t*_2_ = 80 ms) and mid-deceleration (*t*_3_ = 180 ms). Overall, the pressure drops increased during the acceleration phase reaching peak values, decreased by 70% at peak flow and were close to 0 mmHg at the end of systole. Pressure recovery was observed at peak flow and mid-deceleration in all models, whereas at peak systole only the Magna_Pledgets model presented non-uniform pressure distribution in the annulus region. The latter showed lower pressure values in this region (i.e., 15 mmHg), in contrast to the sutureless models (i.e., 17 mmHg) which presented a gradual pressure decrease along the main flow direction.

**Figure 6:**
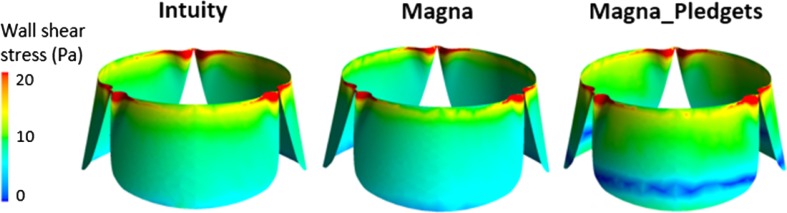
Wall shear stress (WSS) contours at peak systole (*t*_1_ = 40 ms) on the internal surfaces of the leaflets, with Magna_Pledgets valve displaying a low WSS region at the base.

Peak systolic wall shear stress on the leaflets internal surfaces showed a similar pattern in the three valves (Fig. 6), with WSS concentration at the commissures and on the leaflets free edges (maximum values were 41.8–52.4, 37.7–55.3 and 40.1–46.5 Pa for Intuity, Magna and Magna_Pledgets, respectively). However, the Magna_Pledgets valve displayed a low WSS region at the leaflet base (min WSS = 0.86 Pa, being about 94% lower than the WSS observed at the free edge).

**Figure 7:**
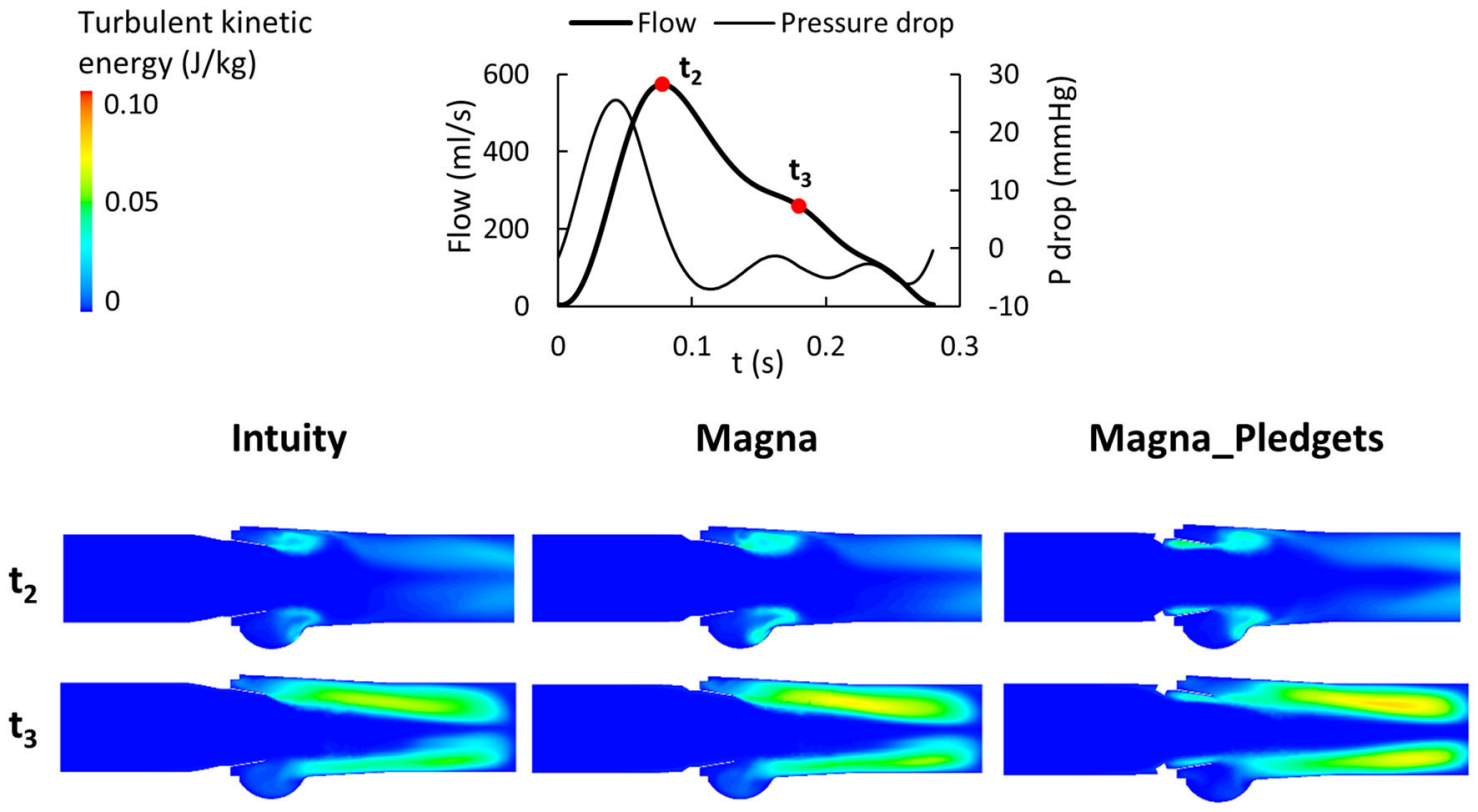
Mapping of turbulent kinetic energy displayed in the axial cross-sections of the three models at peak flow (*t*_2_ = 80 ms) and mid-deceleration (*t*_3_ = 180 ms).

The resulting peak Reynolds and Womersley numbers were about 30,000 and 31, respectively, for all the valves. This indicated that the investigated flow fields were likely characterized by turbulence. Therefore, we computed the TKE in the three valve models at peak flow and mid-deceleration. Overall, TKE was approximately zero in the entire 3D domain except for the valve region and/or the leaflet wake. The greatest TKE values (0.07–0.08 J/kg) were reported at mid-deceleration in all the models. In Fig. 7, TKE is plotted on axial cross-sections. Once again, Intuity and Magna valve models presented similar patterns, whereas the Magna_Pledgets showed higher TKE (x13) in the region adjacent to the internal surface of the leaflets, as well as greater values (i.e., 24%) in the leaflet wake.

**Figure 8:**
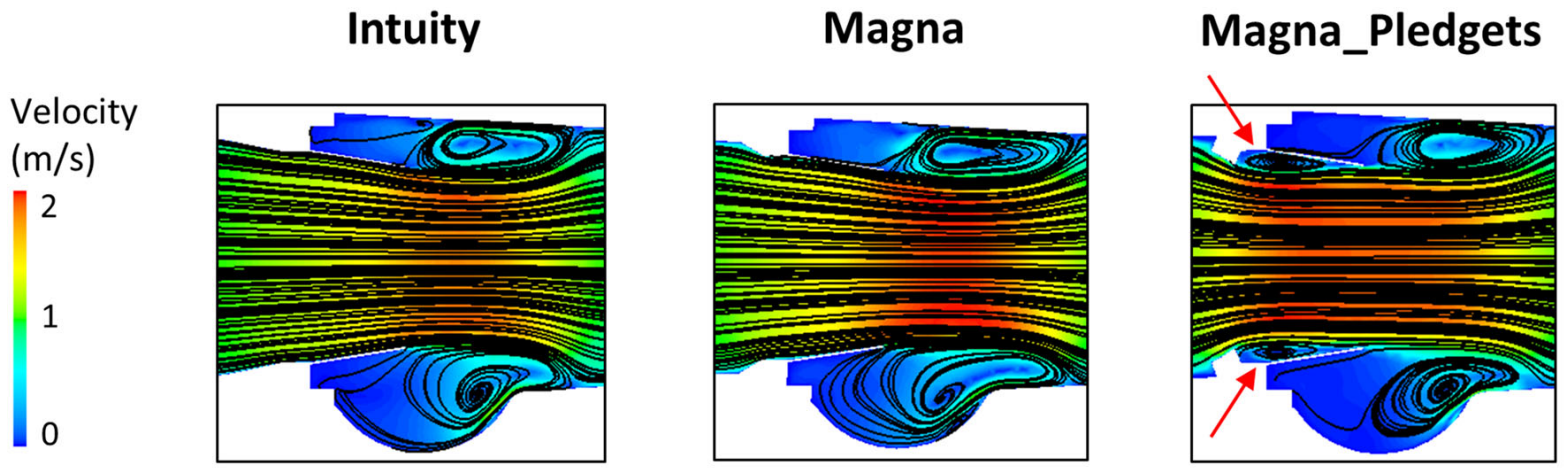
Details of the velocity fields with flow pathlines in axial cross-sections of the three models at peak flow (*t*_2_ = 80 ms). Small vortices and recirculation zones were identified at the internal leaflet surfaces of the Magna_Pledgets valve (red arrows).

By analysing the velocity field through the valves, it is possible to observe in all models a wide central flow jet with eddies formation between the open leaflets and the sinuses during the acceleration phase, and its propagation downstream at peak flow and mid-deceleration. In addition, small vortices and recirculation zones at the internal leaflet base of the Magna_Pledgets valve were noticed during the entire cycle, as magnified by the flow pathlines on the annulus plane (Fig. 8). Although backflow velocity magnitude was small, this never occurred in the sutureless valve models, indicating that pledget-armed sutures played a unique role in determining the flow field.

The presence of flow disturbance and recirculation in the Magna_Pledgets model was also revealed by the number of residual injected particles and by their RT. The number of residual particles with RT >0.1 s, i.e., one-third of the simulated cycle, was higher in the Magna_Pledgets model than in the others (606 vs. 425 and 307 in the Intuity and Magna model, respectively) and their RT was overall longer.

**Figure 9:**
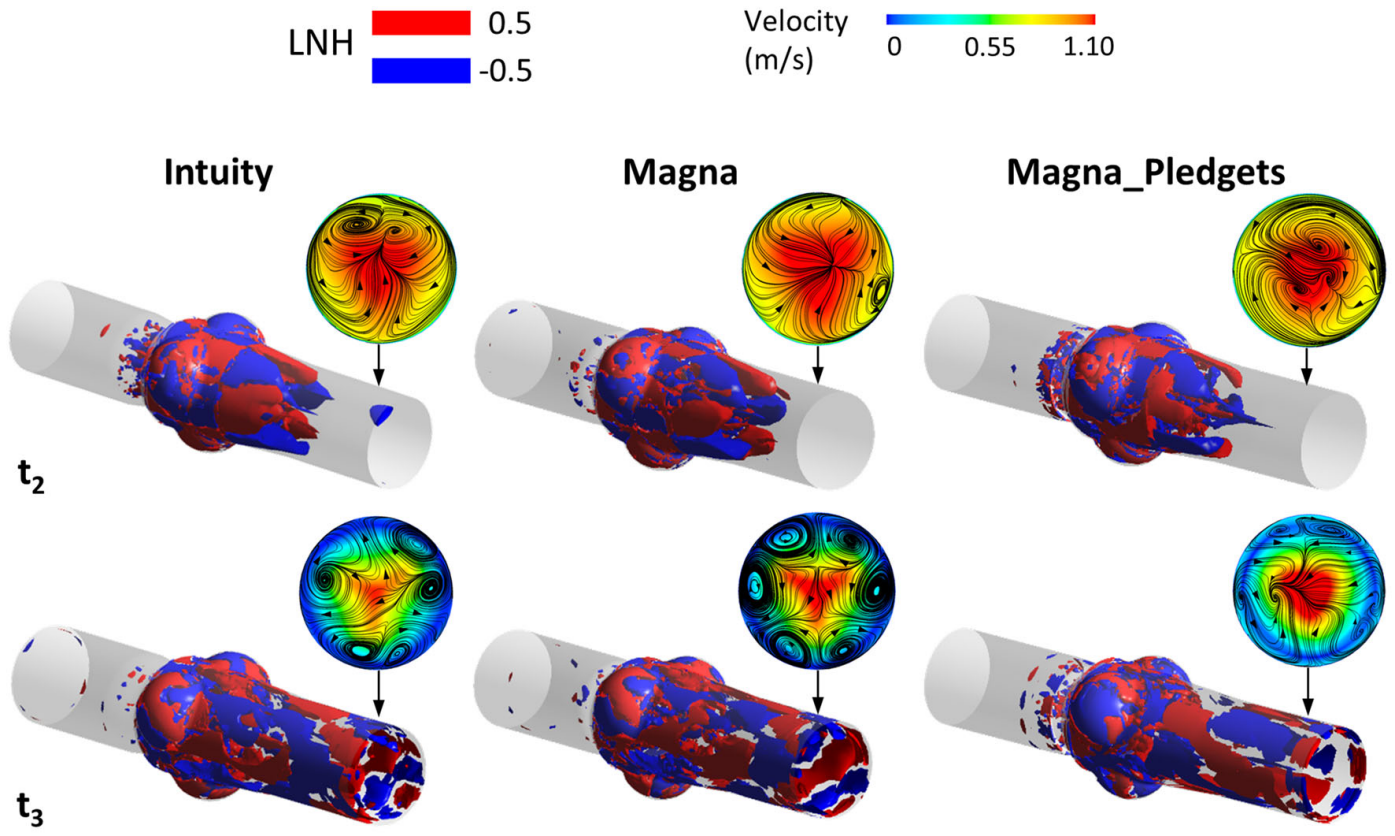
Iso-surfaces of localized normalized helicity at peak flow (*t*_2_ = 80 ms) and mid-deceleration (*t*_3_ = 180 ms). Axial velocity contours and in-plane pathlines are displayed on transverse cross-sections downstream the valves.

Figure 9 displays iso-surfaces of localized normalized helicity (LNH) at peak flow and mid-deceleration. Bulk flow across the Intuity and Magna valves was characterized by counter-rotating fluid structures with moderate helicity intensity, as shown by the positive and negative LNH values (±0.5) of the iso-surfaces. Such fluid structures were well defined at peak flow in both models, with two counter-rotating structures located at each leaflet position, whereas disorganization of the flow field appeared during the deceleration phase. Furthermore, complex flow fields, characterized by secondary flows with different patterns between the three valve models, were highlighted by the in-plane flow pathlines on transverse cross-sections downstream the valve (Fig. 9). In the Magna_Pledgets model flow patterns differed from those of the other models mainly at mid-deceleration, when the flow field was described by fewer and smaller sized eddies.

## Discussion

This study aimed to investigate the effects on the fluid dynamics of different anchoring systems of aortic valve prostheses. In particular, the pledget-armed sutures were isolated in order to quantify their role on the performances of specific bioprostheses. Such assessment was carried out by comparing two devices commercially available: the Carpentier-Edwards PERIMOUNT Magna Ease valve and its rapid-deployment evolution, the Edwards Intuity valve system. These valves, based on identical functional components (i.e., leaflets and supporting frame), were selected among different commercially available valves to exclude the influence of any effects due to different valve constructions and materials. The results confirm that the pledgets-armed sutures can negatively affect the haemodynamic performance of the valve substitutes. Hence, a system which includes a reduced number of pledgets might be preferable. The comparison was performed with a combined approach of experimental and computational analyses with a twofold objective: measuring the different performance and investigating the reasons for such alteration.

Overall, a synergetic approach, combining experiments and numerical simulations, can be advantageous to thoroughly test existing and novel devices. On the one hand, the experimental set-up, i.e., the pulse duplicator system, allowed inferring the baseline of the device properties according to the standards prescribed. These data are usually neither available in the literature nor from the manufacturers, especially for novel devices. They are crucial to implementing a realistic computational model, which is able to replicate standard operative conditions. On the other hand, CFD analyses could enrich the results of the experimental campaign by providing insights on the local fluid-dynamics that can potentially explain the reasons for different valve performance.

It is well known that the fluid-dynamic performance of a bioprosthesis depends on frame design, type and position of the leaflets.^7^,^17^,^30^ However, the recent introduction of rapid-deployment valves has highlighted the potential hemodynamic improvements related to the absence of sutures. Clinical studies comparing rapid-deployment and conventional sewed-in aortic heart valves reported differences regarding MPG and EOA, seemingly confirming this positive tendency.^1^,^16^ However, the currently available clinical studies do not directly compare different techniques, as they are either retrospective and nonrandomized or include different prostheses, and are therefore open to potential biases. In addition, the measurements of the isolated effect of different anchoring systems *in vivo* may only be possible in large and strictly defined patient cohorts. Hence, a combination of *in vitro* and *in silico* tools was here considered to address such a question on the complex flow dynamics.

Gold standard experiments were used, first of all, to quantify fluid-dynamic characteristics by means of a certified pulse duplicator system. Overall, the results of the tests showed excellent fluid dynamic performance of both valves platforms (Magna and Intuity), which were tested according to the ISO 5840 standard. In this context, all three tested configurations exceeded the minimum performance requirements (i.e., EOA larger than 1.0 cm^2^ and regurgitant fraction smaller than 10%). These findings were also in accordance with an analogous study reported in the literature. In particular, the study by Marquez et al.^21^ reported very similar findings with regards to a valve predecessor of the Magna valve tested in this study (i.e., Carpentier-Edwards PERIMOUNT). However, the *in vitro* assessment showed how the different anchoring systems can result in a marked difference between sutured and rapid-deployment valves, with the first one showing significantly lower values of EOA and higher pressure gradients and regurgitant fractions, assessed in all the tested conditions. Also, it was shown how the performance of the Intuity is similar to the Magna valve, if tested without pledget-armed sutures. This comparison also confirmed the hypothesis that the identical design of frame and leaflets corresponds to the equivalent fluid dynamic performance. These results strengthened the choice of including these two valves in this study. Pledgets are essential to securing the valve within the aortic root in supra-annular position. For this reason, it was fundamental to include them in the experimental set-up. However, we used special sutures with low-profile pledgets to reflect our current clinical practice. Results may be more pronounced with different suture types. Our results suggested the importance of closely reproducing the post-implant configuration, whereas possible, for the accurate determination of the valve performance.

Computational fluid dynamics analyses were set up in order to replicate the experimental flow conditions when the valve leaflets were fully open, and thus to investigate the local fluid-dynamics variations in a realistic manner. In this context, the geometry of the apparatus was accurately replicated *in silico* by combining direct measurements, for aortic root and pledgets, and images acquired with a high-speed camera for capturing the leaflets in the open configuration. The implementation of a turbulence model was driven by the experimental conditions, which revealed high values of Reynolds and Womersley numbers. The fluid used for the CFD analyses was characterized by physical properties comparable with those of the saline solution employed in the experiments. In such conditions, the fluid reached a peak velocity of around 1.35 m/s, which is certainly higher than the values observed *in vivo* (aortic peak velocity = 0.9/1 m/s leading to peak Reynolds numbers of 8000/10,000). Although the CFD working conditions were not physiological, we aimed at replicating those of the experimental tests as accurately as possible. By reproducing only the systolic phase, turbulence was likely to occur in most of the simulated cycle. In fact, in order to have a laminar flow under pulsatile conditions with a Womersley number of 31, the Reynolds number should not exceed approximately 7000. This implies that fluid velocity is <0.3 m/s, which in our case holds for only 30% of the simulated cycle. Hence, we chose to apply the SST *k*–*ω* turbulence model as allowing good convergence when both laminar and turbulent flows may be present in the same fluid domain, provided that proper mesh density and *Y* ^+^ values near the wall are used. To this end, we previously performed a mesh sensitivity analysis and ensured that the near-wall *Y* ^+^ values were sufficiently small (i.e., <4).

Overall, the results of the simulations showed a trend in agreement with the experiments. They confirmed the link between geometry and fluid dynamics with the presence of pledgets modifying the performance of the valve. As expected, the main difference between computational and experimental results was found in the calculation of the mean pressure drop, as computer simulations did not take into account the transient phases of opening and closing of the leaflets. In contrast, the calculation of peak pressure differences showed a good agreement (average difference <10%) of the values quantified with both *in vitro* and *in silico* analyses. Moreover, timing of the computational flow and pressure drop tracings well replicated the *in vitro* test. In that, peak flow was observed at 40 ms after peak systole in both cases, in line with the results by Sirois and Sun.^28^ The evaluation of the local fluid dynamics quantities displayed a comparable behaviour of the Intuity and Magna valves, as reported in the experimental tests, and was in agreement with results from the literature. WSS on the leaflet surfaces and TKE distributions throughout the 3D domain reflected those obtained from previous studies, which investigated the hemodynamics of heart valve prostheses using computational models.^11^,^28^ However, TKE values calculated in the present study were greater than those reported in the literature^18^ owing to the higher Reynolds number characterizing the present flow field. The identification of eddies in the sinuses was consistent with findings of experiments which implemented particle imaging velocimetry techniques.^13^ By comparing our results from the valve models with and without sutures, interesting findings can be highlighted. First, it was found that the presence of pledgets leads to non-uniform distribution of pressure and WSS, which may have long-term deleterious effects on valve function. Such considerations will require to be correlated with durability data not yet available. Secondly, TKE mapping confirmed that all the valves introduce or amplify the effects of turbulence and that deceleration causes phases of flow instabilities.^18^ Moreover, in the Magna_Pledgets model, an augmented turbulence was observed in the proximity of the valve leaflets. This increase might explain the different WSS distribution. Third, the LNH iso-surfaces enabled the visualization of complex flow patterns. Namely, the presence of helical flow structures made it possible to observe flow stabilization and further disorganization of the flow field downstream of the valves. This finding proved that helicity contributes to kinetic energy transport reduction from the inertial to the dissipation scale, thus mitigating the transitional effects and promoting flow stabilization.^23^ Nevertheless, within the Magna_Pledgets model LNH iso-surfaces were more disorganized than those in the other two models. This led to mild formation of eddies during the deceleration phase, and to a lower flow stabilization compared with the sutureless valve models. Finally, the higher RT of the particles released in the Magna_Pledgets model, along with the eddies developed at its leaflet base, revealed more considerable flow disturbance and recirculation, which may be contributing in turn to platelet aggregation and thrombosis.

Importantly, findings of this study are limited from a computational perspective as a purely CFD approach simulated only the systolic phase, with the valve leaflets in a fixed open configuration. Furthermore, the CFD model did not take into account the compliance of the implantation sites, which is instead included in the experimental set up. Even if the trend of experimental and computational results is analogous, these simplifications can likely explain the absolute differences of the results obtained, suggesting the implementation of a fluid–structure interaction (FSI) model if a complete validation of the numerical method is to be attempted. However, for the specific purpose of this study, we opted for a CFD methodology to focus our comparison on the different local fluid-dynamics associated with altered anchoring systems. A similar approach was followed by Sirois and colleagues to correlate the local haemodynamics to the risk of blood damage.^28^ The combination of computational models with experimental findings should compensate for this current limitation. The recent advances brought by FSI^4^,^6^,^9^,^20^,^37^ with the integration of different computational techniques will be nevertheless explored in future studies. Finally, only one size of device (i.e., 23 mm) was tested in this study to represent an average size. A wider experimental and computational campaign should therefore be performed to generalize the results of this study.

## Conclusions

Pledget-armed sutures negatively affect the performance of cardiac valve substitutes. The performance of rapid-deployment valves has been confirmed to be non-inferior to their traditional counterparts. A combined approach of experiments and numerical simulations can be effectively used to quantify the relationship between local fluid-dynamics and overall performance associated with different valve technologies.

## References

[1] Andreas M, Wallner S, Habertheuer A, Binder T, Rosenhek R, Wiedemann D (2016). Conventional versus rapid deployment aortic valve replacement: a single center comparison between the Edwards Magna valve and its rapid deployment successor. Interact. CardioVasc. Thorac. Surg..

[2] Ansys. Fluent V15 Documentation, 2015.

[3] Bavaria JE, Desai ND, Cheung A, Petracek MR, Groh MA, Borger MA, Schaff HV (2014). The St Jude Medical Trifecta aortic pericardial valve: results from a global, multicenter, prospective clinical study. J. Thorac. Cardiovasc. Surg..

[4] Bavo AM, Rocatello G, Iannaccone F, Degroote J, Vierendeels J, Segers P (2016). Fluid–structure interaction simulation of prosthetic aortic valves: comparison between immersed boundary and arbitrary Lagrangian–Eulerian techniques for the mesh representation. PLoS ONE.

[5] Bluestein D, Rambod E, Gharib M (2000). Vortex shedding as a mechanism for free emboli formation in mechanical heart valves. J. Biomech. Eng..

[6] Borazjani I, Ge L, Sotiropoulos F (2010). High-resolution fluid–structure interaction simulations of flow through a bi-leaflet mechanical heart valve in an anatomic aorta. Ann. Biomed. Eng..

[7] Bottio T, Caprili L, Casarotto D, Gerosa G (2004). Small aortic annulus: the hydrodynamic performances of 5 commercially available bileaflet mechanical valves. J. Thorac. Cardiovasc. Surg..

[8] Carabello BA (2009). Aortic stenosis. Lancet.

[9] Chandra S, Rajamannan NM, Sucosky P (2012). Computational assessment of bicuspid aortic valve wall-shear stress: implications for calcific aortic valve disease. Biomech Model. Mechanobiol..

[10] Claiborne TE, Sheriff J, Kuetting M, Steinseifer U, Slepian MJ, Bluestein D (2013). In vitro evaluation of a novel hemodynamically optimized trileaflet polymeric prosthetic heart valve. J. Biomech. Eng..

[11] Claiborne TE, Xenos M, Sheriff J, Chiu WC, Soares J, Alemu Y, Gupta S, Judex S, Slepian MJ, Bluestein D (2013). Towards optimization of a novel trileaflet polymeric prosthetic heart valve via device thrombogenicity emulation (DTE). ASAIO J..

[12] Cosgrove DM, Sabik JF (1996). Minimally invasive approach for aortic valve operations. Ann. Thorac. Surg..

[13] Ducci A, Tzamtzis S, Mullen MJ, Burriesci G (2013). Hemodynamics in the Valsalva sinuses after transcatheter aortic valve implantation (TAVI). J. Heart Valve Dis..

[14] Dumont K, Vierendeels J, Kaminsky R, Van Nooten G, Verdonck P, Bluestein D (2007). Comparison of the hemodynamic and thrombogenic performance of two bileaflet mechanical heart valves using a CFD/FSI model. J. Biomech. Eng..

[15] Flameng W, Herregods M-C, Hermans H, Van Der Mieren G, Vercalsteren M, Poortmans G (2011). Effect of sutureless implantation of the Perceval S aortic valve bioprosthesis on intraoperative and early postoperative outcomes. J. Thorac. Cardiovasc. Surg..

[16] Kocher AA, Laufer G, Haverich A, Shrestha M, Walther T, Misfeld M (2013). One-year outcomes of the surgical treatment of aortic stenosis with a next generation surgical aortic valve (TRITON) trial: a prospective multicenter study of rapid-deployment aortic valve replacement with the Edwards Intuity Valve System. J. Thorac. Cardiovasc. Surg..

[17] Kuehnel R-U, Puchner R, Pohl A, Wendt MO, Hartrumpf M, Pohl M, Albes JM (2005). Characteristic resistance curves of aortic valve substitutes facilitate individualized decision for a particular type. Eur. J. Cardiothorac. Surg..

[18] Les A, Shadden S, Figueroa CA, Park J, Tedesco MM, Herfkens RJ, Dalman RL, Taylor CA (2010). Quantification of hemodynamics in abdominal aortic aneurysms during rest and exercise using magnetic resonance imaging and computational fluid dynamics. Ann. Biomed. Eng..

[19] Mareels G, Kaminsky R, Eloot S, Verdonck PR (2007). Particle image velocimetry-validated, computational fluid dynamics-based design to reduce shear stress and residence time in central venous hemodialysis catheters. ASAIO J..

[20] Marom G, Haj-Ali R, Raanani E, Schäfers HJ, Rosenfeld M (2012). A fluid–structure interaction model of the aortic valve with coaptation and compliant aortic root. Med. Biol. Eng. Comput..

[21] Marquez S, Hon RT, Yoganathan AP (2001). Comparative hydrodynamic evaluation of bioprosthetic heart valves. J. Heart Valve Dis..

[22] Menter FR (1994). Two-equation eddy-viscosity turbulence models for engineering applications. AIAA J..

[23] Moffatt HK, Tsinober A (1992). Helicity in laminar and turbulent flow. Annu. Rev. Fluid Mech..

[24] Morbiducci U, Gallo D, Cristofanelli S, Ponzini R, Deriu MA, Rizzo G, Steinman DA (2015). A rational approach to defining principal axes of multidirectional wall shear stress in realistic vascular geometries, with application to the study of the influence of helical flow on wall shear stress directionality in aorta. J. Biomech..

[25] Nishimura RA, Otto CM, Bonow RO, Carabello BA, Erwin JP, Guyton RA (2014). 2014 AHA/ACC guideline for the management of patients with valvular heart disease: executive summary. J. Am. Coll. Cardiol..

[26] Phan K, Tsai Y-C, Niranjan N, Bouchard D, Carrel TP, Dapunt OE (2015). Sutureless aortic valve replacement: a systematic review and meta-analysis. Ann. Cardiothorac. Surg..

[27] Rahmani B, Tzamtzis S, Ghanbari H, Burriesci G, Seifalian AM (2012). Manufacturing and hydrodynamic assessment of a novel aortic valve made of a new nanocomposite polymer. J. Biomech..

[28] Sirois E, Sun W (2011). Computational evaluation of platelet activation induced by a bioprosthetic heart valve. Artif. Organs.

[29] Tabata M, Shibayama K, Watanabe H, Sato Y, Fukui T, Takanashi S (2014). Simple interrupted suturing increases valve performance after aortic valve replacement with a small supra-annular bioprosthesis. J. Thorac. Cardiovasc. Surg..

[30] Tasca G (2015). Does the type of suture technique affect the fluid-dynamic performance of bioprostheses implanted in small aortic roots? Results from an in vitro study. J. Thorac. Cardiovasc. Surg..

[31] The International Standard Cardiovascular implants—Cardiac Valve Prostheses (ISO 5840: 2009). 7952303

[32] Ugur M, Suri RM, Daly RC, Dearani JA, Park SJ, Joyce LD, Burkhart HM, Greason KL, Schaff HV (2014). Comparison of early hemodynamic performance of 3 aortic valve bioprostheses. J. Thorac. Cardiovasc. Surg..

[33] Ugur M, Byrne JG, Bavaria JE, Cheung A, Petracek M, Groh MA (2014). Suture technique does not affect hemodynamic performance of the small supra-annular Trifecta bioprosthesis. J. Thorac. Cardiovasc. Surg..

[34] Vahanian A, Alfieri O, Andreotti F, Antunes MJ, Barón-Esquivias G, Baumgartner H, Borger MA, Carrel TP, De Bonis M, Evangelista A, Falk V (2012). Guidelines on the management of valvular heart disease (version 2012). Eur. Heart J..

[35] Verdonck P (2002). The role of computational fluid dynamics for artificial organ design. Artif. Organs.

[36] Wollersheim LW, Li WW, de Mol BA (2014). Current status of surgical treatment for aortic valve stenosis. J. Card. Surg..

[37] Wu W, Pott D, Mazza B, Sironi T, Dordoni E, Chiastra C, Petrini L, Pennati G, Dubini G, Steinseifer U, Sonntag S (2016). Fluid-structure interaction model of a percutaneous aortic valve: comparison with an in vitro test and feasibility study in a patient-specific case. Ann. Biomed. Eng..

[38] Xenos M, Girdhar G, Alemu Y, Jesty J, Slepian M, Einav S (2010). Device thrombogenicity emulator (DTE)—Design optimization methodology for cardiovascular devices: a study in two bileaflet MHV designs. J. Biomech..

